# Anomaly Detection in Endemic Disease Surveillance Data Using Machine Learning Techniques

**DOI:** 10.3390/healthcare11131896

**Published:** 2023-06-30

**Authors:** Peter U. Eze, Nicholas Geard, Ivo Mueller, Iadine Chades

**Affiliations:** 1 School of Computing and Information Systems, The University of Melbourne, Parkville, VIC 3010, Australia; nicholas.geard@unimelb.edu.au; 2 Walter and Eliza Hall Institute of Medical Research, Parkville, VIC 3052, Australia; mueller@wehi.edu.au; 3 CSIRO, Ecosciences Precinct, Dutton Park, QLD 4102, Australia; iadine.chades@csiro.au

**Keywords:** anomaly detection, malaria, machine learning, big data

## Abstract

Disease surveillance is used to monitor ongoing control activities, detect early outbreaks, and inform intervention priorities and policies. However, data from disease surveillance that could be used to support real-time decisionmaking remain largely underutilised. Using the Brazilian Amazon malaria surveillance dataset as a case study, in this paper we explore the potential for unsupervised anomaly detection machine learning techniques to discover signals of epidemiological interest. We found that our models were able to provide an early indication of outbreak onset, outbreak peaks, and change points in the proportion of positive malaria cases. Specifically, the sustained rise in malaria in the Brazilian Amazon in 2016 was flagged by several models. We found that no single model detected all anomalies across all health regions. Because of this, we provide the minimum number of machine learning models *top-k* models) to maximise the number of anomalies detected across different health regions. We discovered that the top three models that maximise the coverage of the number and types of anomalies detected across the thirteen health regions are principal component analysis, stochastic outlier selection, and the minimum covariance determinant. Anomaly detection is a potentially valuable approach to discovering patterns of epidemiological importance when confronted with a large volume of data across space and time. Our exploratory approach can be replicated for other diseases and locations to inform monitoring, timely interventions, and actions towards the goal of controlling endemic disease.

## 1. Introduction

Disease surveillance programs established by local, state, and national governments collect health data of potential epidemiological importance [1]. The volume and velocity of data collected through these systems are increasing over time [2]. Despite the increasing availability of disease surveillance data, much of it is not used adequately or optimally [2] to support real-time public health decisionmaking. Identified reasons for this underutilisation of epidemiological data include limited availability of data analysts, data quality issues such as delayed and missing data, and competing priorities for health system resources [3,4,5]. As a consequence, opportunities to use surveillance data to inform timely public health decisionmaking are being missed. Here, using malaria as a case study, we explore the benefits of using anomaly detection to inform timely decisionmaking about interventions with the aim of controlling disease transmission [6].

Depending on the type of disease outbreak, different methods of generating evidence from surveillance data may be useful in supporting public health decisionmaking. In outbreak scenarios, a simple dashboard of case numbers and locations may be sufficient to inform daily estimates of growth and the estimated size and timing of peaks, informing decisions about interventions. However, for endemic diseases the ongoing analysis of case reports by humans may not be frequently performed. As regular insights may be useful to inform the use of interventions, methods that enable automated generation of evidence from surveillance data are required.

Several forms of evidence have been identified as being useful to inform decision-making around control of endemic disease, including estimating disease burden in a population, identifying when, why, and how with respect to rapid growth in case numbers beyond a given threshold (flareups), identifying spatiotemporal variations in progress towards disease elimination, and estimating the effectiveness of interventions [7]. Most of the evidence required for endemic disease management can be detected as deviations from the known characteristics of the endemic disease recorded in the surveillance data. The departure from normal behaviour and the process of detecting such departures by mining the collected data is termed anomaly or outlier detection [8,9,10].

An anomaly is an observation that deviates so much from other observations as to arouse suspicion that it was generated by a different mechanism [10] or as an error in the current mechanism. Anomaly detection is the process of mining patterns in data that do not tally with the expected behaviour of the system that generates the data [9]. Across a variety of different types of system, anomalies may occur as a result of reporting errors, variability in measurement, change in natural processes, human-induced errors, faults in machines, fraudulent activities, and response to intervention measures, among other reasons.

In this paper, we use a variety of anomaly detection methods to detect anomalies in endemic disease surveillance data, with the purpose of assisting public health managers to make better decisions. The reason for employing different detection methods is due to the variations in data distribution from one health region to another. In this way, the best method for a given health region would eventually be deployed. The objectives of this paper include: (i) designing an anomaly detection framework that enables the repeatable integration of methods, data, and generated insights into the decision support system of different regions to assist in the control and eventual elimination of endemic diseases; (ii) evaluating the potential epidemiological significance of the spatio-temporal anomalous patterns detected by the machine learning models; and (iii) assessing the consistency and variation in anomalous patterns detected by models in order to select the best models to be deployed into production for anomaly detection in surveillance datastreams.

With the above objectives in mind, we sought to answer the following research questions. (1) What are the relevant epidemiological anomalies that can be detected in routine disease surveillance data? (2) What is the minimum number of models (*top-k* models) that should be deployed to maximise the number of detected anomalies across different health regions? (3) How consistent and variable is the performance of these models across the data from different health regions?

We used historic data on malaria incidence in the state of Para in the Amazon region of Brazil, where malaria remains endemic. The surveillance data for this case study consist of a de-identified and public version of the Brazilian epidemiological surveillance system of malaria (SIVEP-Malaria) database recorded from 2009 to 2019 [11]. This dataset contains daily positive and negative test results, aggregated into months in this work as the days and dates have been removed. The state of *Para* is divided into thirteen health regions for health administration purposes.

In the remainder of this paper, we first introduce our framework for exploring anomaly detection in surveillance data, then provide details of the dataset used to validate this framework. We describe the various unsupervised machine learning algorithms that we employed for exploring anomalies in the data. We then present the results of our analysis and discuss the relevance of our framework and anomaly detection methods more broadly in the context of the control and management of endemic diseases.

## 2. Methods

In this section, we first introduce our design of a framework to enable automated and repeatable surveillance data analysis for anomaly detection irrespective of space and time. We then leverage the components of the framework to describe how the data in the case study were processed and different unsupervised anomaly detection algorithms were applied to detect patterns and anomalies of different types.

### 2.1. Anomaly Detection Framework

A major consideration in designing a modern data analysis framework is taking data drift into consideration [12,13]. Data drift refers to the changes in data distribution over time and space. Continuously integrating new data and retraining the anomaly detection algorithm is important to ensure that the performance of a deployed anomaly detection model remains up to date.

**Figure 1:**
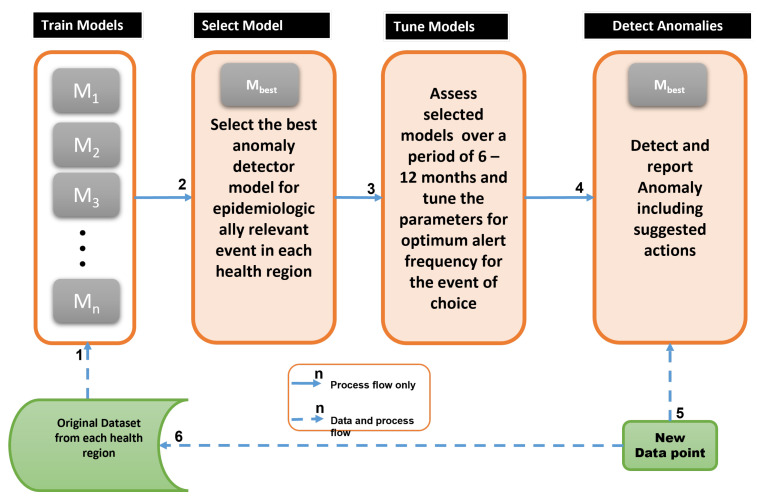
Anomaly detection framework, showing process and surveillance data flow; $M_{1}$ to $M_{n}$ refer to ensembles of anomaly detection models. The appropriate definition of an anomaly for each health region is used to select the best anomaly detector ($M_{best}$) for that health region. The models are retrained and tuned over time as novel data becomes available.

Figure 1 presents a decision support framework that is based on anomaly detection using endemic disease surveillance data. In endemic disease, we assume that case numbers and situation reports are not analysed on a daily basis by humans. This assumption is in contrast with ongoing pandemics such as COVID-19 [14], in which daily case analysis is carried out. Hence, only a monthly review triggered by alerts would lead to conscious analysis and investigation by humans. Daily reports take an average of one month to be collated and aggregated into the health regions that make up a state government. The entirety of the surveillance data for a given health region is used as a training set for the anomaly detection models.

To understand the framework in Figure 1, Table 1 provides a description of each of the steps. The framework can be used to consistently apply anomaly detection models to surveillance data collected over time in different regions and for different endemic diseases.

The remaining subsections provide further details on the data transformations, feature extraction procedure, and machine learning algorithms used for training the anomaly detection models along with the major parameters used by the training algorithms.

### 2.2. Epidemiological Feature Selection and Preprocessing

Although there are 42 fields recorded in the SIVEP time series surveillance data for malaria [11], for the purposes of this study we used four fields: date (in months), the total number of tests, number of negative results, and number of positive results.

These last three features were converted into a single feature, the *proportion of positive tests* $I_{P}$, which is the proportion of tests that were conducted that returned a positive result for malaria. The conversion was conducted for each state and health region. $I_{P}$ is mathematically defined as

(1) $$I_{P}=\frac{N_{P}}{N_{T}}$$

where $N_{P}$ is the total number of positive cases per month and $N_{T}$ is the number of tests carried out per month. As $I_{P}$ is a proportion, we can compare values across time and space even if the testing capacity changes over months and across geographical health regions. However, we assume a uniform distribution of cases across a health region such that there is an equal chance of detecting an infected person within a given health region.

With the assumed uniform distribution of positive cases per health region, an increase in $I_{P}$ would then truly represent the situation where more people in the health region are becoming affected, and the reason for the rise can then be investigated. Considering the same testing capacity ($N_{T}$), a decline in $I_{P}$ would then represent either a naturally declining epidemic situation or the outcome of a deployed intervention.

**Figure 2:**
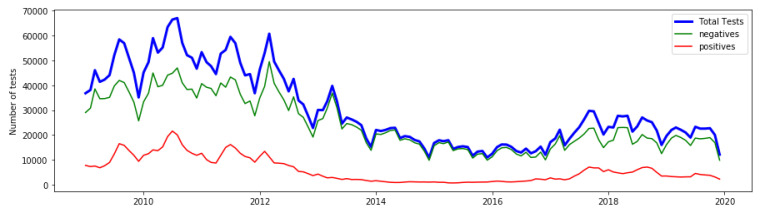
Original recorded surveillance data from the state of Para, Brazil, showing the monthly total tests (blue), number of negatives (green), and number of positives (red).

Figure 2 shows the state-level aggregated data from the state of Para, Brazil which we use here to demonstrate the original epidemiological features of interest used to derive $I_{P}$.

**Figure 3:**
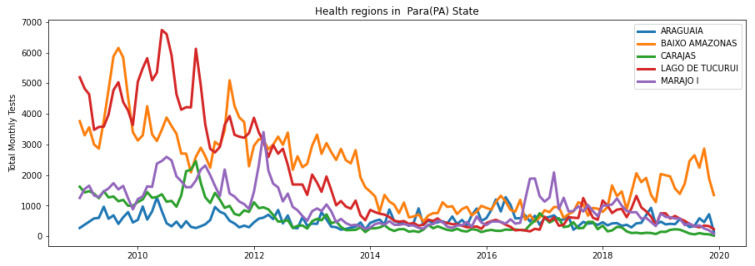
Total monthly tests per health region (five of thirteen regions are shown here for brevity); the data represent $N_{T}$ in Equation (1) for each health region.

Similar data to those in Figure 2 were extracted for each of the thirteen health regions by separating Para into its different health regions. The plots for five of the thirteen regions are shown in Figure 3.

**Figure 4:**
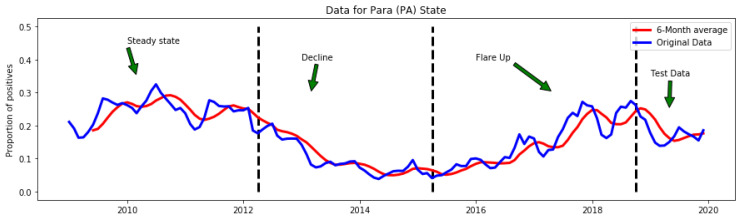
Feature engineering and time series data transformation. Endemic outbreaks can move from a steady state to periods of either rapid growth (flareup) or decline in $I_{P}$.

The extracted data were then transformed to $I_{P}$ for each of the health regions. An example outcome of the data transformation is shown in Figure 4. To reduce noise, we applied the moving average transformation to the derived feature $I_{P}$ using a window size of six months. The moving average is used to remove noise and irregularity (e(t) in Equation (2)) to enhance the prediction accuracy of the machine learning algorithms.

Time series data y(t) can be generalised using the following additive model:

(2) y(t)=g(t)+s(t)+h(t)+e(t)

where

g(t) is the trend (change over a long period of time)

s(t) is seasonality (periodic or short-term changes)

h(t) represents the effect of holidays on the forecast

e(t) is the error term or irregularities (specific unconditional changes).

Depending on which components of time series are present in the data, different learning algorithms can be used to model time series data.

The unsupervised approach to anomaly detection is exploratory in nature, and evaluation is subjectively performed by humans. In medicine and epidemiology, machine learning and anomaly detection systems are meant to assist doctors and epidemiologists when data volumes become overwhelming. For unsupervised methods, there is no ground truth to train the algorithm; therefore, the output of such systems needs to be validated and interpreted by humans while taking the disease and its epidemiological parameters into consideration. We used this approach in evaluating the dates flagged as anomalies by different models in this research.

Although unsupervised anomaly detection algorithms are provided with certain inputs by humans that enable them to set a metric threshold for objectively and automatically detecting anomalies, the detected anomalies need to be certified by experts. Table 2 shows the unsupervised models which are integrated into the Pycaret framework [15], using a specific distance measure to estimate the point anomalies in time series data.

**Clustering-based local outlier** (cluster or CBL), local outlier factor (lof), and connectivity-based local outlier (cof) are based on local outlier concepts. CBL uses a distance measure that considers both the distance of an object to its nearest cluster and the size of the cluster; thus, small clusters are not simply discarded as outliers. The *lof* algorithm uses a k-nearest neighbour approach to define the density of objects, with their distances from one another then considered in defining the density of the locality. The *reachability distance*, which is a non-symmetric measure of distance, is used to determine an outlier. Each datapoint may have a different reachability distance, and this distance is used to define the degree of anomaly. The larger the value, the more anomalous the point from its local neighbours [16].

**Connectivity-based local outlier** *cof* is an improved version of *lof*. The density-based lof algorithm has a shortcoming in that it completely depends on the density of the neighbouring points. It performs poorly when the density of the outlier is similar to its nearby datapoints. Hence, *cof* recognises that anomalies must not be of a lower density than the data from which they deviate [17].

**Isolation Forest** (iforest) algorithm is an unsupervised version of decision tree. It uses a binary decision tree. The iforest algorithm is based on the assumptions that anomalies are few and unique and that they belong to the shallow branches where they easily isolate themselves from the rest of the tree branches. A random set of features are selected and used to build the tree using the datapoints. A sample that travels deep into the tree is unlikely to be anomalous. The Isolation Forest algorithm is computationally efficient and is very effective in anomaly detection; however, the final anomaly score depends on the *contamination parameter* provided while training the model, meaning that some idea of what percentage of the data are anomalous is required to reach a better prediction [18].

**Histogram-based anomaly detection** (histogram) assumes feature independence and builds the histogram of each feature. The anomaly score is based on the histogram-based outlier score (HBOS). The HBOS can be constructed from either univariate or multivariate features of each datapoint. For multivariate problems, the anomaly score of all variables is added up to rank the data. Starting with *d* variables having *p* datapoints, the HBOS is calculated as [19]

(3) $$HBOS\left(p\right)=\sum_{i=0}^{d}log\left(\frac{1}{hist_{i}\left(p\right)}\right).$$

Histogram outlier detection first constructs a histogram for a variable by choosing a bin. The computed score for each variable is normalised to 1.0 and summed across the variables *d* to compute the global outlier score. A datapoint may be anomalous in one variable and non-anomalous in others; hence, a data point that is an outlier in almost all the variables is almost definitely an anomaly in the dataset.

**K-Nearest Neighbors** (KNN) is popularly used as a supervised learning algorithm. However, it can be used as an unsupervised learning algorithm as well, and can be used to detect outliers or anomalies in data. The assumption in this implementation for anomaly detection is that outliers are not in close *proximity* to other neighbours. A threshold is defined for proximity and used to determine datapoints that do not belong to a neighbourhood. A key parameter that determines the number of neighbours to be used in calculating the proximity measure is the *n_neighbors* parameter.

**One-class SVM detector** (svm) is an unsupervised version of a traditional SVM regressor or classifier. It uses either a min-volume hypersphere [21] or a max-margin hyperplane metric to separate anomalous data from the normal ones. The major purpose of one-class SVM is to detect novelties in data, which helps to detect rare events. Novelty and weak signals are special aspects of anomaly detection. In one-class SVM, datapoints that lie outside the hypershere or below the hyperplane are considered anomalies.

**Principal Component Analysis** (PCA) is a method that decomposes a signal into its major components. The first component is usually the most important. This is followed by the second, third, etc. The idea of using PCA for outlier detection is that a datapoint with a high reconstruction error from its principal components is an outlier from the dataset [22]. For different PCA algorithms, the way the anomaly score is calculated may differ. The use of residuals, leverage, and influence of a datapoint may all be put into consideration. However, these metrics are better utilised in a visualisation than in an automated outlier detection system. Hence, human evaluation and domain knowledge may need to be applied in setting the threshold for outlier detection using the appropriate metrics for the problem domain.

**Minimum Covariance Determinant** (MCD) is an anomaly detection method that uses the fact that tightly distributed data have a smaller covariance determinant value. Thus, instead of using the entire dataset to calculate distribution parameters (such as mean and standard deviation), it divides the data into subsamples and then computes the covariance determinant of each subgroup. The number of subsamples *h* is such that $\frac{n}{2}<h<n$, where *n* is the total number of datapoints [23]. The group with minimum covariant determinant is used as the central group for distance calculation. This approach is best suited for determining outliers in multivariate data [23]. MCD uses *robust distance* measures that are not amenable to the unrealistic distributional assumptions that underlie the use of *Mahalanobis distance* measures for outlier detection in most other classical methods. Mahalanobis distance computation is sensitive to the presence of outliers in data, as the outliers tend to draw the distributional statistics towards themselves. Hence, the robust distance is a robust calculation of the *Mahalanobis distance* such that the effect of outliers is minimised. Other alternatives or variations for *Mahalanobis distance* when dealing with outlier detection for high-dimensional data include the comedian approach [25] and robust Mahalanobis distance with shrinkage estimators [26]. We do not consider our data to be high-dimensional, and do not explore this topic in the present research.

**Stochastic outlier selection** (SOS) [24] is a statistical modeling method for anomaly detection. This method assumes that the data follow a stochastic model with a form of probability density function (pdf). Normal data exist in areas of higher density, while anomalies exist in areas of lower density. Hence, the measure of distance used to determine anomaly is the *probability density*. For parametric stochastic models, a pdf is assumed a priori, with values assumed for the model parameters. However, for non-parametric modelling few or no assumptions are made about the value of these parameters, and the algorithm has to learn the model parameters directly from the data. We have followed largely non-parametric modelling in this work. We focus on discovering the model that best models the data based on the type of anomaly that is of interest to epidemiologists. In this research, we are interested in *outbreak anomalies*.

A major parameter and assumption that underlies the algorithms and methods employed in this work, which are based on unlabelled data, is the use of the *proportion of anomaly* or *contamination rate*, η. The contamination rate is the fraction of the total data that we assume to be anomalous. Our default value is 0.1 (10%) of the data. In our standard experiments, we set η=0.1. In addition, we conducted a sensitivity analysis for this parameter using values of η=[0.1,0.2,0.3,0.4].

### 2.3. Selecting the Top-*k* Models for Maximum Coverage

The problem of selecting models that optimise both anomaly detection coverage and inference time (finding the *top-k model*) is a version of the classical set cover and maximum coverage problems [27]. The set cover problem is described as follows: consider a universe U and a family S of subsets of U, where a cover is a subfamily K⊆S of sets with a union of U. In simple terms, and in this research, the set cover problem involves finding the lowest number of models that can detect the same number of anomalies detected by the ten models. In situations where the top *k* models cannot detect all of the possible anomalies, the maximum coverage problem seeks to ensure that no other set of models *k* can detect more anomalies than the selected top *k* models. Hence, we want to select the top *k* models to maximise anomaly detection.

In this work, we gradually increased coverage until *k* models were selected or 100% coverage was achieved. At each stage, we chose a model with a detected anomaly set $K_{i}$ which contained the largest number of uncovered elements. We repeated this process until 100% coverage was achieved up to a maximum of *k* times. We then recorded either the model set that achieved 100% coverage or the *top-k* models and the percentage coverage they achieved. We performed this analysis for each health region and for all health regions combined into a single unit.

## 3. Results

In this section, we present the major results relating to the algorithms and the discovered anomalies of interest.

### 3.1. Anomalies by Contamination Rate

The number of anomalies detected by each algorithm is linearly proportional to the contamination rate η. This observation is consistent across all thirteen health regions. The anomalous datapoints were subjected to evaluation by epidemiological experts to interpret their significance. As the contamination rate increases, more datapoints are flagged as anomalies. The contamination rate that identifies all the anomalies of interest to epidemiologists is retained and deployed for anomaly detection within that health region. In the next subsection, we describe the nature and location of the detected anomalies.

### 3.2. Detected Anomalies and Epidemiological Significance

For the purpose of this study, we define epidemiological events of interest as the onset of an outbreak, the peaks and troughs of an outbreak, and change points in the proportion of positive cases. Models that distinctly flagged these events were considered to perform better than others. Generally, we first state the classification of a detected anomalous point before proceeding to the second phase involving the use of multiple models to detect the same anomaly. Thus, in practice, the evaluation process is iterative.

**Figure 5:**
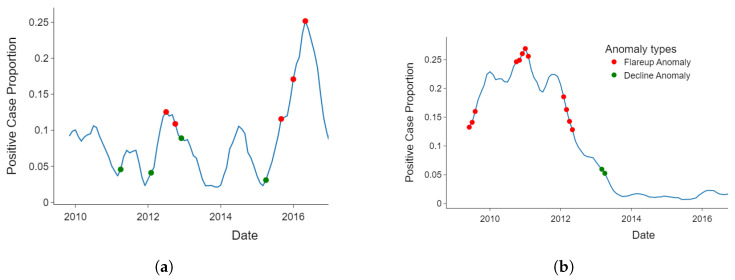
Early change-point anomalies vs. collocated anomalies: (**a**) early flareups detected (Araguaia health region, detected by sos model) and (**b**) clusters of anomalies excluding early flareups (Xingu health, region detected by cof model).

Figure 5 shows the anomalies detected from Araguaia and Xingu. Figure 5a shows that the early increase or decline of cases can be detected from the data. Thus, the change points in the datasets are detected as anomalies. This is appropriate for either early detection of outbreaks or early signs of the effectiveness of an intervention as indicated by declining incidence.

Figure 5b shows the continuous rising points, peak points, and continuous falling points detected as anomalous. These anomalous clusters show a strong change in the curve direction. This type of anomaly detection is suitable for reducing false alarms. An alarm related to anomalous points is only triggered when a change in the direction of the data has been strongly established.

The results in Figure 5a,b were respectively produced by the stochastic outlier selection (SoS) and cluster-based models. Therefore, health regions with low risk tolerance could implement the SoS model, while more risk-tolerant health regions could implement cluster-based anomaly detection.

**Figure 6:**
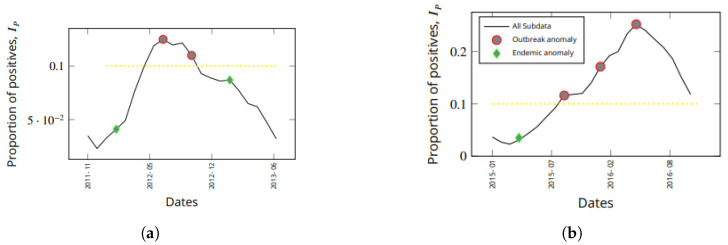
For early detection of endemic disease transitions, the sos algorithm is more appropriate. The yellow dashed line represents the threshold for separating anomalies that occur within outbreaks (when the positivity rate is more than or equal to the 10% of the sampled population) and endemic situations (when the positivity rate is less than 10% of the sampled population). (**a**) Early detection of a small outbreak and (**b**) early detection of a large outbreak.

It is worth understanding how early an outbreaks can be detected. The *outbreak detection time* is the time between the first report of a rising epidemic and the time that the model flags a report as anomalous. Another important time of note is the time between the first detection of an increasing outbreak and the peak of the outbreak. This later time determines how effective a deployed intervention can be when an anomaly is detected. Figure 6 shows results from the Araguaia health region, illustrating how early the two major outbreaks over the past ten years could have been detected. In this figure, we have zoomed in on the relevant portions of Figure 5a to illustrate early flagging of outbreaks. We have chosen Araguaia as it has experienced numerous outbreaks throughout the decade of 2009 to 2019.

In Figure 6a, the first instance of an increasing proportion of positive cases ($I_{P}$) was reported in January 2012. As the next report in February 2012 arrived, these data were reported as being anomalous. Thus, only one time step delay occurred before anomaly detection. This outbreak peaked in July 2012, five months after it was detected. There were no further alarms between February and July 2012, meaning that the flareup trend continued.

In Figure 6b, there was a larger outbreak. In April 2015, the first occurrence of high case prevalence was immediately flagged as anomalous. Hence, there was zero delay in flagging that very early phase of an outbreak as being anomalous. No further alarm was triggered until September 2015, and then until January 2016, until the outbreak peaked in May 2016. While the alert in September 2015 can be explained as a possible slowdown in the outbreak, that of January 2016 indicates a continued rise in the proportion of positive cases. These are strategic anomalous alerts, which can be very useful in planning interventions or in investigating why an existing interventions is not being successful.

In both examples shown in Figure 6a, it is clear that outbreaks can be detected very early (with a lag time of zero or one time step) using the appropriate anomaly detection algorithm that is most suitable for modeling early detection in a given health region.

### 3.3. Consistency and Variation in Anomalies Detected by Models

**Figure 7:**
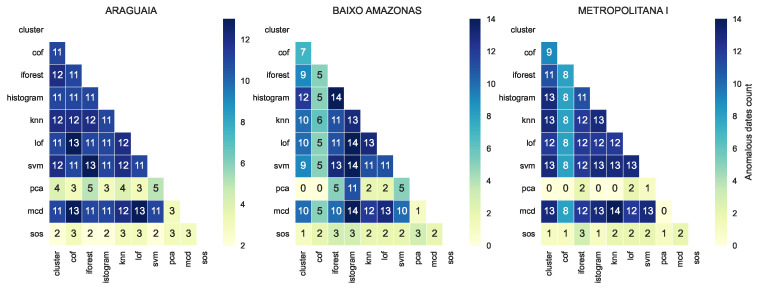
Each cell in the heat map shows the anomalies detected jointly by two models. The models *pca* and *sos* have strong disagreement both with each other and other models as to the dates flagged as anomalous.

For the dates on which the proportion of positive cases was detected as anomalous, we checked how many of the models jointly detected such dates as anomalous. We plotted a heat map of the number of anomalous dates jointly detected by model pairs. Certain patterns can be seen to emerge within and across health regions, as shown in Figure 7. We highlight which models mostly agreed with other models and which models rarely agreed.

The pattern shows that a majority of the models found the same points to be anomalous. Specifically, *cluster*, *cof*, *forest*, *histogram*, *knn*, *lof*, *svm*, and *mcd* agreed on at least 85% of their detected anomalous datapoints. In contrast, *pca* and *sos* disagreed with all other models and with each other on the datapoints marked as anomalous. Models that are jointly consistent their prediction or detection may be used as an ensemble to cross-validate anomalous datapoints, which can increase the confidence of decision-makers. Other models that vary considerably in their prediction or disagree with other models should not be discarded, as these models are important in detecting novelty, rare events, early changes, or weak signals.

**Figure 8:**
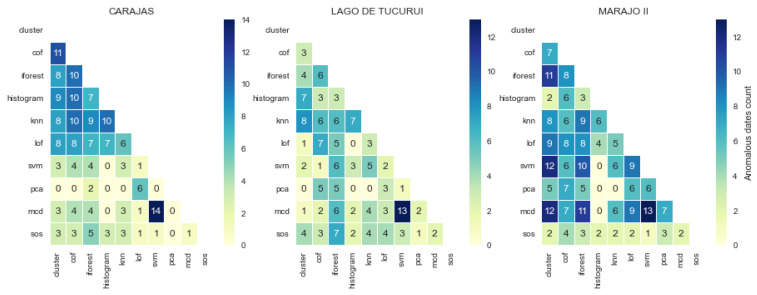
Increasing variation in jointly detected anomalies in certain health regions.

In other health regions, several new patterns and changes to previously described patterns were observed, as shown in Figure 8. In these cases, most models detected different types of anomalies, and did not agree in as many cases as previously.

Most of the jointly consistent models continued to detect the same type and number of anomalies, though to lesser extent. It can be observed that *svm* and *mcd* disagreed with other models that they were previously consistent with, and strongly agreed with each other in detecting the same number of anomalies. In Carajas, all the data points flagged as anomalous by *svm* were flagged as anomalous by *mcd* as well. In Lego de Tucuri, both models had thirteen out of the fourteen anomalous points they detected in common. In Lego de Tucuri, however, it is difficult to establish strong consistency among models. It seems that all models detected largely disjoint anomalous points. As inconsistencies continued to wane across all models, *sos* showed even stronger disagreement with other models (see Marajo II) in terms of the type of jointly detected anomalies.

It can be inferred that even though different models detect similar anomalies, the distribution of each dataset from different regions is likely to change the behaviour of each model, resulting in a different type and number of anomalies detected in each region.

Even without detailed reference to the mathematical details underlying these models, we can identify similar models irrespective of data distribution. Next, we examine the temporal variations in the detected anomalies.

### 3.4. Temporal Location of Detected Anomalies

**Figure 9:**
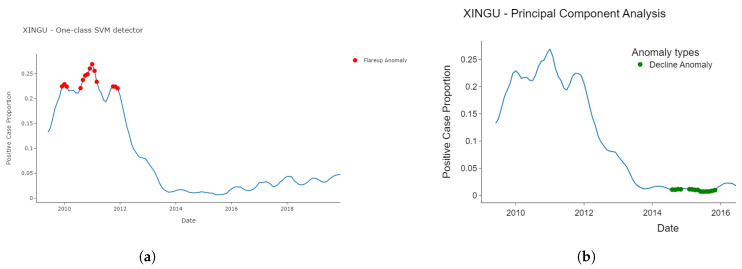
The location of anomalies detected by different algorithms varying by the year in which they were detected. Events occurring in these years in a given health region may help to interpret the significance of anomalies detected in that region. (**a**) Outbreak anomalies located by SVM between 2011 and 2014 and (**b**) decline anomalies located by PCA between 2015 and 2016.

We observed that the temporal location of the anomalies flagged by the models were significantly different, despite most of the models flagging an almost equal number of anomalies per contamination rate. Figure 9 exemplifies the location of the detected anomalies by several algorithms distributed across different months and years.

Figure 9 clearly shows the variation in the temporal locations of anomalies detected by two algorithms. The One-Class SVM (Figure 9a) and PCA (Figure 9b) algorithms provide interesting and contrasting anomalies. Whereas the first detected the peaks of the outbreaks, the other detected the troughs of the declining epidemic. Hence, the attention of human experts may be required to validate the output of each model and then select the best model for each region based on the type of anomalous event most relevant to that region.

**Figure 10:**
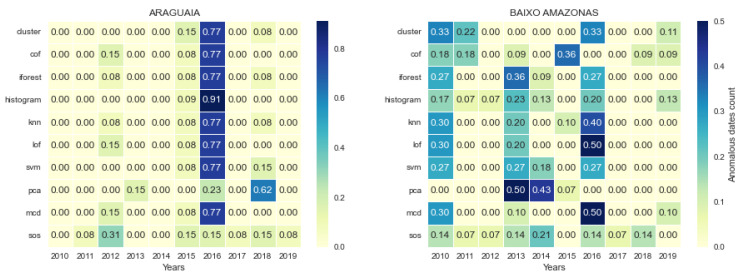
Proportion of anomalies per model per year. There is a clustered temporal location (Araguaia) and a dispersed temporal location (Baixo) of anomalies.

In order to compare the locations of detected anomalies across models, time, and space, we defined a parameter called the *proportion of anomalies per year*. This is the ratio of anomalies detected by a model in a year to the total number of anomalies detected over a decade; hence, the values lie between 0 and 1. Figure 10 shows two major variations in the temporal locations of the detected anomalies across regions.

In Figure 10 (Araguaia), eight of the ten models detected 77% or more anomalies in 2016 alone. This is a very strong agreement among the models irrespective of the type of anomaly detected by each model. This provides strong evidence in support of further investigation about what happened in 2016 regarding the epidemiology of malaria in this region in comparison with other years. Hence, we can say that the anomalies in this health region were clustered in 2016. We observed similar situations in Carajas (2011), Metropolitana I (2010), Metropolitana III (2010), and Rio Caetes (2010).

A dispersed temporal location of anomalies was observed in certain regions, as no single year had large number of anomalies detected. However, certain models co-detected anomalies in certain years. For example, in Baixo Amazonas a significant number of models (at least six out of ten) detected more than 20% of anomalies in each of 2010, 2013, and 2016. Again, 2016 has strong outlier content. This is because *knn*, *lof*, and *mcd* detected more than 40% anomalies in 2016 alone when compared to the nine other years under consideration.

The importance of the anomalies detected by the models can be seen from the perspective of more models detecting the same anomaly within certain temporal domain or few models detecting few anomalies within a temporal domain. Each of these behaviours may prove either that a known anomalous event occurred within a temporal location or that a novelty was being detect by only a few models.

### 3.5. Model Selection for Inclusion in Endemic Disease Surveillance System

For practical purposes, the thirteen health regions under study will not deploy and maintain as many models as the ten employed in this study. It would be financially and technically demanding for the state government to maintain ten different models across thirteen health regions. Moreover, several of the models detected redundant anomalies, with as many as eight models jointly detecting one anomaly. Although this redundant detection helps to increase confidence in the detected anomalies, it increases the inference time in production. Hence, after establishing consistency in detected anomalies across models, we propose selecting only the top *k* models that provide either 100% coverage of all the anomalies or a predetermined high percentage (75–95%) of all anomalies.

**Figure 11:**
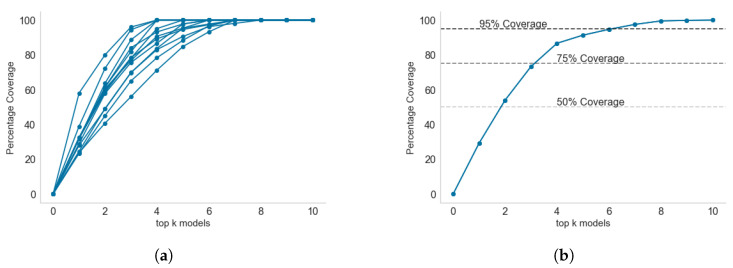
Local and global coverage by the *top-k* models: (**a**) between four and eight models are required to achieve 100% coverage per health region; (**b**) anomaly coverage of *top-k* when anomalies from the thirteen health regions are combined. Six models achieved 95% coverage across all thirteen health regions. (**a**) Local coverage by *top-k* models and (**b**) global coverage by *top-k* models.

To solve the above problem, we applied the classical set cover and maximum coverage greedy heuristic algorithm [27]. The results showing the progression of percentage coverage as more models are added to the *k* subsets are shown in Figure 11.

Across the thirteen health regions, between four and eight models are required to achieve 100% coverage per region. This is known as the local coverage, as only the anomalies detected in each region are fed as input to the set cover algorithm. For global coverage analysis, Figure 11b shows that all models are required to achieve 100% coverage. However, the first few models have a high percentage of anomaly coverage. Only two models, *pca* and *sos*, are required to cover 50% of all the anomalies in the thirteen health regions. Three models can achieve 73.53% coverage, while six models can achieve 94.81% coverage. With 95% coverage, there is good coverage of all possible anomalies without implementing the last four models, though without considering which models are locally relevant to each health region.

**Figure 12:**
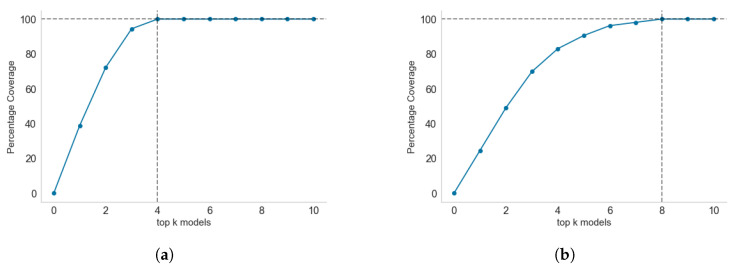
Progression of coverage in selected health regions with minimum (four) and maximum (eight) model sets required to achieve 100% anomaly coverage: (**a**) smallest number of models required to achieve 100% coverage, with four models required and *cluster* and *sos* together achieving 77% coverage; (**b**) largest number of models required to achieve 100% coverage, with eight models and the first four models achieving about 80% coverage.

With reference to individual health regions, Figure 12 shows the coverage progression as more models are added to a health region. We have used Araguaia (Figure 11a) and Tocantins (Figure 11b) to illustrate the regions that required the smallest number of models to achieve 100% coverage and the largest number of models to achieve 100% coverage, respectively.

It is interesting to see how the local ranking of models differs from a global ranking of models. The top-ranked models locally differ from the global ranking. For example, the *top three* global models (Figure 11b) are *pca*, *sos*, and *mcd*, covering 73.53% of all anomalies. In contrast, the *top three* local models (in Araguaia) are *cluster*, *sos*, and *pca* covering about 97%. In Tocantins, the *top three* models are *cluster*, *pca*, and *cof*, covering only 72.2% of anomalies detected in the region. Only *pca* consistently ranked among the *top three* models in both the global and local coverage problems. The *cluster* algorithm only ranked sixth in the global coverage problem, while it ranked first in the two local coverage problems. This result shows that whereas *cluster* may have detected most anomalies in Araguaia and Tocantins, the reverse is the case for most of the other eleven regions under consideration.

## 4. Discussion

The availability of large amounts of public health data is not currently matched by their use to support real-time public health decisionmaking [4]. Methods and frameworks are needed that can use historical data to discover patterns and provide insights to support decision-making for disease control. In this work, we have explored ten unsupervised machine learning models and evaluated their potential to discover anomalies in malaria surveillance data. In addition, we have designed a framework that enables continuous integration of new data to update decision support in near-real-time.

We used models to detect three anomalous patterns relevant to epidemiology: rapid growth in cases (*flareup*), drastic *decline* in cases, and *change in trend direction* of the proportion of positive cases. These events and patterns were chosen on the basis of having been deemed epidemiologically important in previous work [28,29]. We found that the *one-class svm* model was the model best able to detect the peaks of outbreak flareups, while *pca* was best able to detect the valley of decline in the proportion of positive cases. The change in the direction of positive case trends was best detected by the *sos* model. Considering that model performance varied across health regions, a standard framework was adopted to allow new data to be analysed in a reproducible way using all available models.

We found that no single method or model performed well in detecting all predefined anomalies across all health regions. When all of the thirteen health regions in Para state were combined, *pca*, *sos*, and *mcd* were found to be the top three models that maximised the number and types of anomalies detected. However, in individual health regions, the *cluster* algorithm ranked higher than *pca* and *sos* in terms of maximising the number of anomalies detected when used alone. Overall, *pca* and *sos* performed well on average both across individual health regions and for the combined health regions.

These results can provide guidance about model selection when focusing either on a specific type of anomaly or on maximising the broad range of anomalies detected, and when we are focusing on either a specific health region or on the combination of regions at the state level. The choice of which combination of models to use is likely to be driven by the risk appetite of a health region and the type of epidemiological anomaly they are most interested in detecting and mitigating. For risk-averse regions, models that detect rare events may be included even though few other models confirm this rare event. Health regions with higher risk appetite might focus on models that confirm well-known anomalies. For example, model ensembles (*svm*, *mcd*, *cluster*) that confirm that an outbreak has actually taken off can be deployed regions with higher risk appetites, while models that detect early outbreaks such as *sos* could be deployed in risk-averse regions even when no other model confirms an alert for early warning against a potential outbreak.

There are two methods of controlling alarm fatigue considered in this work, namely, true positives and false alarms. The first seeks to reduce the contamination rate parameter for each anomaly detector. The second approach relies on the confirmation of an alarm event by all *top-k* models before a warning alarm is sent out. In the second case, *k* is the set of models that determines the same type of anomalies more often than not. Hence, model selection depends on their consistency in detecting the anomaly of concern over time. The limit in the number of models to be selected and deployed is affected by the space and time complexity as well [30]. The number of alarms sent per unit time and the critical nature of an alarm determine how fatigued a human recipient becomes.

The pipeline in Figure 1 can be replicated in different settings with different surveillance data to select the best model for anomalies of different kinds ranging from flareups, declines, and changes to the direction of epidemic curves. Further, it serves as a simple experimental framework that is compliant with continuous integration and continuous deployment (CI/CD) paradigm [13]. Considering the dynamic nature of disease outbreaks, a CI/CD-compliant framework should take care of both data drift and concept drift [31,32] and continually select the best model as new data arrives. Concept and data drift may be experienced within a health region over time and may be encountered when a model trained using data from one region is deployed into another region or for different disease surveillance data.

This work has several limitations and possibilities for future extension. As unsupervised anomaly detection methods do not use labeled event classification, input from domain experts is required to help validate their predictions. New patterns are first identified from the data and plausible interpretations are provided afterwards. Hence, the epidemiological significance assigned to different anomalies detected in the endemic data remains subject to further expert evaluations. Again, although we assumed that the distribution of the data changes over time, we did not quantify the magnitude of the drift in order to formally and dynamically determine when model swap or retraining should be triggered. Future work will focus on performing drift analysis and formalising the drift threshold [12,31] for warning alerts and automatic model selection, retraining, or replacement.

In conclusion, this paper has demonstrated that anomaly detection models can be successfully applied to epidemiological surveillance data to discover unknown patterns that are relevant for intervention design and formulation of disease elimination strategy. However, only the *top-k* models that maximise the detection of the anomaly of concern should be deployed and maintained in production. This approach strikes a balance between the detection of all anomalies and the cost in resources required to run multiple models to maximise the type of anomalies detected.

The volume and variety of public health data have rendered many statistical methods inadequate for extracting evidence for robust decision-making. For example, sources of public health information such as patient health records as well as other digital traces such as social media, blogs, internet documents, phone logs, and recorded voice logs cannot be adequately analysed using statistical methods [33]. In general, unsupervised machine learning methods such as anomaly detection models can utilise a larger volume of surveillance data to provide deeper insights to better support decision-making for the elimination of endemic diseases.

## Figures and Tables

**Table 1:** Summary of the pipeline methods and steps for online anomaly detection using surveillance data.

Step No.	Major Activities
1	Train candidate anomaly detectors per health region using train set
2	Based on local epidemic demands, select best anomaly detector, $M_{best}$
3	Tune model parameters after using for 6–12 months to evaluate performance
4	As new data arrives, use the best detector, $M_{best}$ to detect and interpret anomaly
5	New data for evaluation and model re-training
6	Update the models with the new data and repeat from Step 1

**Table 2:** Unsupervised anomaly detection algorithms. The distance measures used by each algorithm differ. A single anomaly score is computed for each datapoint. Based on a contamination rate (10% by default), a threshold anomaly score is used to flag anomalous datapoints.

No.	Model ID.	Model Name	Core Distance Measure
1	cluster	Clustering-Based Local Outlier [16]	Local outlier factor
2	cof	Connectivity-Based Local Outlier [17]	average chaining distance
3	iforest	Isolation Forest [18]	Depth of leaf branch
4	histogram	Histogram-based Outlier Detection [19]	HBOS
5	knn	K-Nearest Neighbors Detector [20]	Distance Proximity
6	lof	Local Outlier Factor [16]	Reacheability distance
7	svm	One-class SVM detector [21]	hyper-sphere volume
8	pca	Principal Component Analysis [22]	Magnitude of reconstruction error
9	mcd	Minimum Covariance Determinant [23]	Robust distance from MCD
10	sos	Stochastic Outlier Selection [24]	Affinity probability density

## Data Availability

Code and derived datasets generated during the study can be found at https://github.com/KingPeter2014/Anomaly_in_malaria_surveillance_data.

## Abbreviations

The following abbreviations are used in this manuscript:

AI	Artificial Intelligence
PCA	Principal Component Analysis
pdf	Probability Density Function
SVM	Support Vector Machine
